# Treatment of Goitre by Iodine

**Published:** 1890-01-11

**Authors:** 


### TREATMENT OF GOITRE BY IODINE.

Mr. Terillon has met with great success in the treatment of goitres by injections of iodine, which lead to fatty degeneration and absorption of the gland tissue and its replacement by tough newly-formed connective tissue. In some instances a single injection of eight or ten drops have effected a cure, in others as many as twenty injections repeated at intervals of four or six days have been necessary. The dangers to be feared are suppuration and the direct access of the solution to the circulation: the former may be avoided by boiling the syringe for several minutes before use, thus effectually sterilising it, and the latter by allowing the canula to remain in the gland for some moments after withdrawing the trocar, when, if any, blood exude, indicating the wound of a vessel, it must be introduced at another point.

